# Timing of menarche and menopause and epigenetic aging among U.S. adults: results from the National Health and Nutrition Examination Survey 1999–2002

**DOI:** 10.1186/s13148-025-01827-x

**Published:** 2025-02-21

**Authors:** Saher Daredia, Dennis Khodasevich, Nicole Gladish, Hanyang Shen, Jamaji C. Nwanaji-Enwerem, Anne K. Bozack, Belinda L. Needham, David H. Rehkopf, Julianna Deardorff, Andres Cardenas

**Affiliations:** 1 Division of Epidemiology, School of Public Health, University of California, Berkeley, USA; 2 Department of Epidemiology and Population Health, School of Medicine, Stanford University, Stanford, CA 94305 USA; 3 Department of Emergency Medicine, Perelman School of Medicine, University of Pennsylvania, Philadelphia, USA; 4 Center of Excellence in Environmental Toxicology, Perelman School of Medicine, University of Pennsylvania, Philadelphia, USA; 5 Department of Epidemiology, Center for Social Epidemiology and Population Health, School of Public Health, University of Michigan, Ann Arbor, USA; 6 Division of Community Health Sciences, School of Public Health, University of California, Berkeley, USA; 7 Department of Pediatrics, School of Medicine, Stanford University, Stanford, USA

**Keywords:** Menarche, Menopause, DNA methylation, Epigenetic aging

## Abstract

Reproductive aging, including timing of menarche and menopause, influences long-term morbidity and mortality in women, yet underlying biological mechanisms remain poorly understood. Using DNA methylation-based biomarkers, we assessed associations of age at menarche (*N* = 1,033) and menopause (*N* = 658) with epigenetic aging in a nationally representative sample of women ≥ 50 years. Later age at menopause was associated with lower GrimAge epigenetic age deviation ($B$ = − 0.10 years, 95% CI: − 0.19, − 0.02). No associations were observed for menarche timing. This suggests a connection between earlier menopause and biological aging, with potential clinical implications for identifying those at high risk for age-related disease.

**Supplementary Information:**

The online version contains supplementary material available at 10.1186/s13148-025-01827-x.

## Introduction

Reproductive health is not only critical to women’s overall health and wellbeing, but is increasingly shown to be a key predictor of future chronic disease risk. An earlier age of menarche, defined as first occurrence of menstruation, is associated with earlier all-cause mortality and increased risk of breast and endometrial cancers, diabetes, and cardiovascular disease (CVD) [1, 2]. Research on the long-term impacts of menopausal timing, defined as occurring 12 months after a woman’s last menstrual period, has varied by health outcome, with earlier onset linked to a higher risk of CVD and all-cause mortality, but a lower risk of breast cancer [2, 3].

In the past 60 years, the mean age of menarche has decreased by 0.8 years; while, the mean age of natural menopause has increased by 1.5 years among women in the United States (U.S.) [3]. These shifting trends in reproductive timing may have broader implications for aging processes and long-term health outcomes. Reproductive aging has also been shown to influence overall aging processes in organisms at the cellular, tissue, organ, and system levels [4]. Despite this, there is a notable gap in research exploring the relationship between reproductive timing and epigenetic age biomarkers, which are DNA methylation (DNAm)-based predictors of morbidity and mortality. Referred to as epigenetic clocks, these biomarkers are strong predictors of chronological age and derived from DNAm levels of specific cytosine–phosphate–guanine (CpG) sites [5]. Developed using different human tissue types and age-related outcomes, these clocks capture different aspects of biological aging [5].

There are established associations between many lifestyle and environmental factors and altered epigenetic aging [5]. Only a handful of studies have considered the relationship between menarche and menopausal timing with epigenetic aging in later adulthood. Earlier menarche has been linked to accelerated epigenetic aging in studies of adolescent and pre-menopausal women [6, 7]. Additionally, later menopause has been associated with slower epigenetic aging in a few reports [8, 9]. However, most of these studies are limited by small sample sizes and limited diversity in participant characteristics, reducing generalizability of findings to the broader U.S. population.

In this study, we examine the association of ages at menarche and menopause with epigenetic aging in the National Health and Nutrition Examination Survey (NHANES), a nationally representative sample of adults in the United States that includes demographic, questionnaire, and laboratory data. We hypothesize that earlier timing of these reproductive phases is associated with accelerated epigenetic aging.

## Methods

NHANES is a biannual, nationally representative cross-sectional survey conducted by the National Center for Health Statistics (NCHS) to assess the health of the noninstitutionalized U.S. population [10]. Our study consisted of adult participants ages 50–84 years who participated in the 1999–2000 and 2001–2002 NHANES cycles and provided blood samples for DNAm analysis. After applying eligibility criteria, we analyzed *N* = 1,033 and *N* = 658 participants who had self-reported questionnaire data available on timing of menarche and menopause, respectively. Analyses only included individuals with natural menopause, excluding those whose menopause was induced by medical conditions or treatments. Further details on defining the analytic sample can be found in Supplemental Figs. 1–2. Ages at menarche and menopause were self-reported in the NHANES reproductive health questionnaire which was administered to female-identifying participants [10]. Data on epigenetic age and DNAm-derived blood cell proportion estimates were downloaded from the NHANES website [10]. Our analysis included several epigenetic age measures, including the Horvath, Hannum, SkinBlood, PhenoAge, GrimAge, GrimAge2, DunedinPoAm, and DNAmTL clocks [5]. Epigenetic age deviation was calculated for each clock as the residuals from a linear regression model of chronological age on each epigenetic aging biomarker. Positive epigenetic age deviation (> 0 years) occurs when epigenetic age is greater than chronological age, reflecting accelerated epigenetic aging; meanwhile, negative epigenetic age deviation (< 0 years) indicates epigenetic age is lower than chronological age. DNAmTL, an estimate of leukocyte telomere length, and DunedinPoAm, an estimate of relative pace of aging, were left untransformed in our analysis. Generalized linear regression models with survey weights were used to evaluate associations of ages at menarche and menopause with each epigenetic age deviation measure. Main models were adjusted for chronological age in years (continuous), chronological age in years squared (continuous), and self-identified race/ethnicity. Further details on exposure and outcome assessment, covariates, and sensitivity analyses can be found in the Supplemental Methods.

## Results

Table 1 describes the unweighted characteristics of study participants in the menarche and menopause analytic samples. The majority of participants identified as Non-Hispanic White (40% of both samples), followed by Mexican American (29% of both samples), Non-Hispanic Black (menarche sample: 21%; menopause sample: 19%), Other Hispanic (menarche sample: 6%; menopause sample: 7%), and Other or Multiracial (4% of both samples). Participants in the menarche and menopause samples had a mean (SD) chronological age of 64.9 (9.3) and 66.1 (9.1) years, respectively, at the time of the NHANES screening. In the menarche sample, participants reported reaching menarche at a mean age of 12.9 (1.7) years. In the menopause sample, participants reported experiencing menopause at a mean age of 50.0 (4.9) years. Participants in both samples had a mean body mass index (BMI) of 29.4 (menarche SD: 6.5; menopause SD: 6.3) kg/m^2^ at the time of screening. Most participants reported never smoking (59% of both samples) and abstaining from alcohol (menarche sample: 54%; menopause sample: 53%).

**Table 1:** Unweighted participant characteristics included in analyses of age at menarche and menopause: NHANES 1999–2002. Values represent unweighted count (%) or mean (SD)

	Menarche sample (*N* = 1,033)	Menopause sample (*N* = 658)
Age at screening, years	64.9 (9.3)	66.1 (9.1)
Age at menarche, years	12.9 (1.7)	13.0 (1.7)
Age at menopause, years	49.9 (4.9)*	50.0 (4.9)
*Race/ethnicity*		
Mexican American	300 (29.0%)	193 (29.3%)
Other Hispanic	66 (6.4%)	49 (7.4%)
Non-Hispanic White	410 (39.7%)	264 (40.1%)
Non-Hispanic Black	216 (20.9%)	124 (18.8%)
Other Race/Multi-Racial	41 (4.0%)	28 (4.3%)
*Body mass index (BMI) at screening, kg/m*^*2*^	29.4 (6.5)	29.4 (6.3)
Missing (n)	23 (2.2%)	15 (2.3%)
*Smoking status at screening*		
Ever	418 (40.5%)	265 (40.3%)
Never	612 (59.2%)	391 (59.4%)
Missing	3 (0.3%)	2 (0.3%)
*Alcohol intake at screening*		
Abstainer	557 (53.9%)	350 (53.2%)
Moderate drinker	450 (43.6%)	293 (44.5%)
Heavy drinker	25 (2.4%)	14 (2.1%)
Missing	1 (0.1%)	1 (0.2%)

^*^Available for *N* = 646 of *N* = 1,033 who met the criteria for having undergone menopause

We observed strong positive correlations between chronological age and epigenetic age across all unique 1,045 participants in both samples, ranging from *r* = 0.76 (Median Absolute Error, MAE: 10.7 years) with PhenoAge to *r* = 0.89 (MAE: 2.8 years) with SkinBlood (Supplemental Fig. 3). DNAmTL was negatively correlated with chronological age (*r* = − 0.60), indicating shortening telomere length with increasing age. The DunedinPoAm biomarker was uncorrelated with chronological age, as expected.

**Fig. 1:**
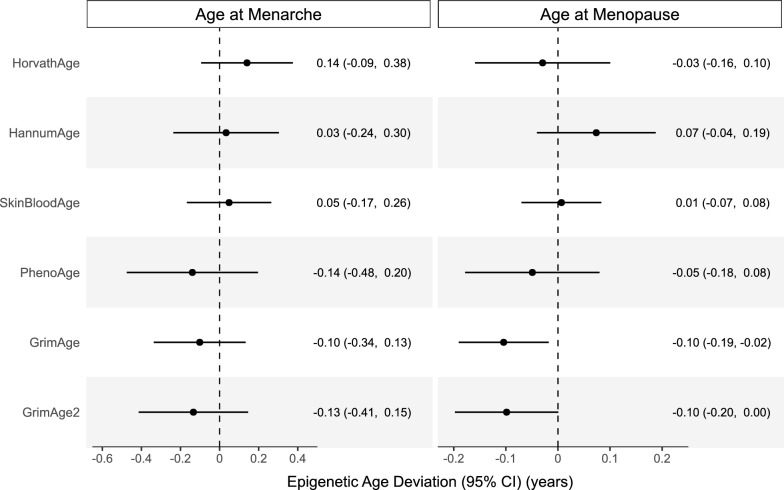
Adjusted estimates and 95% CI for associations of epigenetic age deviation with age at menarche and age at menopause. Associations were assessed using survey-weighted generalized linear regression models. All models were adjusted for chronological age in years (continuous), chronological age squared, and self-identified race/ethnicity. Estimates are regression coefficients ($B$) that represent the estimated change in each epigenetic age biomarker per one-year increase in age at menarche or age at menopause

In primary models adjusted for chronological age, chronological age squared, and self-identified race/ethnicity, we did not observe any associations between age at menarche and epigenetic age deviation across any of the epigenetic aging biomarkers (Fig. 1). Additionally, there were no observed associations between ages at menarche and menopause with untransformed DNAmTL and DunedinPoAm (Supplementary Fig. 4). However, one-year greater age at menopause was significantly associated with lower GrimAge epigenetic age deviation ($B$ = − 0.10 years, 95% CI: − 0.19, − 0.02) at the time of the NHANES screening. A similar trend was observed for GrimAge2, where increased age at menopause was associated with a negative deviation ($B$ = − 0.10 years, 95% CI: − 0.20, 0.00), but this association was imprecise. Given these findings, we examined associations of age at menopause with the DNAm predicted components of GrimAge and GrimAge2. We observed that one-year greater age at menopause was associated with lower DNA methylation estimates of adrenomedullin (ADM) ($B$ = − 0.36, 95% CI: − 0.68, − 0.04) and plasminogen activation inhibitor 1 (PAI1) ($B$ = − 56.70, 95% CI: − 113.22, − 0.19) — both of which are components of the GrimAge and GrimAge2 biomarkers. Associations between age at menopause and epigenetic age deviation from the other clocks were consistently close to the null (Fig. 1). Sensitivity analyses additionally adjusted for (i) BMI, smoking status, and alcohol intake at the time of the NHANES screening and (ii) estimated cell-type proportions showed similar, but attenuated, associations between later age at menopause and negative GrimAge and GrimAge2 epigenetic age deviation (Supplemental Figs. 5–6). We observed no evidence for effect modification by chronological age at blood draw, both in stratified analyses and models with statistical interaction terms.

## Discussion

We examined associations of menarche and menopausal timing with epigenetic aging biomarkers in a representative sample of United States women aged 50–84 years from NHANES. In models adjusted for chronological age and race/ethnicity, we observed that greater age at menopause was associated with lower GrimAge. We did not find any relationship between age at menarche and epigenetic aging.

Our findings on menopausal timing are supported by existing literature. Another study also found an inverse relationship between age at menopause and GrimAge epigenetic aging ($B$=−0.06 years) among women greater than 50 years of age [9]. GrimAge is a composite biomarker that is based on DNAm surrogates of seven plasma proteins linked to morbidity and mortality as well as smoking pack years (i.e., the number of packs of cigarettes smoked per day multiplied by the numbers of years an individual smoked) [9]. Unlike first-generation epigenetic clocks, which were trained to predict chronological age, GrimAge is a robust predictor of time-to-disease and time-to-death [9]. Since GrimAge is associated with disease risk and mortality, the observed association likely reflects the long-term health impacts of menopausal timing which influences biological aging processes and susceptibility to age-related chronic disease. This is supported by our similar findings with GrimAge2, an updated version that leverages additional DNAm-based estimators of plasma proteins and better predicts mortality across multiple racial/ethnic groups. A separate meta-analysis did report similar findings with menopausal timing and Horvath epigenetic aging across three U.S and European-based cohorts—two of which were predominantly non-Hispanic White ($B$= − 0.01 to−0.06 years) [8]. While the overall estimate was significant, associations were not significant among individual cohorts. Notably, similar to our study, two of the three cohorts included women over the age of 50 years, and all three cohorts had a mean age at epigenetic age assessment ranging from 65 to 71 years. This suggests that methodological differences are likely not the reason for the discrepancy in findings.

Nonetheless, the results were in the same direction as those in NHANES, and the findings with the Horvath epigenetic aging biomarker suggest that other biological aging pathways may be involved in menopausal timing, although this was not observed in our sample. In sensitivity analyses, we found that our results on menopausal timing and epigenetic aging were attenuated after adjustment for BMI, smoking, and alcohol intake. While these covariates are potential confounders of the association between menopausal timing and epigenetic aging, they were ascertained at the time of the NHANES screening/sample collection (post exposure) and no retrospective data was available. Since assessment occurred after menopause, it is possible that these covariates are on the causal pathway in the relationship, in which case adjustment may have led to overadjustment bias. We also observed attenuation after adjusting for estimated cell-type proportions, suggesting that the link between menopausal timing and GrimAge may be influenced by inflammatory or other leukocyte-mediated processes that occur as the immune system ages.

Notably, our null findings regarding age at menarche differ from those reported in previous studies. A study of primarily White women based in the San Francisco Bay Area found that later age at menarche was associated with negative GrimAge epigenetic age deviation ($B$= − 0.45 years) among pre-menopausal women ages 25 to 51 years [6]. Another longitudinal study observed that a five-year positive Horvath epigenetic age deviation was associated with significantly earlier age of menarche (Hazard Ratio, HR = 1.29) among Chilean adolescent girls. A possible explanation for the discrepancy between these findings and ours is the more proximal timing between menarche and epigenetic age assessment in these studies. In contrast, the longer time gap between menarche and assessment of epigenetic age in our sample of women aged > = 50 years, coupled with the influence of other exposures that may have altered epigenetic aging trajectories, may have contributed to the lack of a detectable association.

To our knowledge, this is the largest study of menarche and menopausal timing and epigenetic aging in a sample of adults that is representative of the entire U.S. population. Still, our study does have some important limitations. Although we adjusted for demographic and behavioral covariates, there may be residual confounding due to unmeasured covariates such as pre-pubertal and pre-menopausal physical activity, diet, BMI, smoking, and alcohol consumption. Our exposures, ages at menarche and menopause, may also be subject to recall error, but we expect this to be non-differential and bias our results toward the null. Future research should examine longitudinal changes in epigenetic aging before and after the pubertal and menopausal transitions. Additionally, more objective clinical indicators, such as Tanner staging, can be used to better assess the relationship between pubertal timing and epigenetic aging as menarche often occurs later in the pubertal transition. Regardless, our findings do suggest that earlier timing of reproductive phases, particularly menopause, is associated with accelerated epigenetic aging, which may have clinical implications for early identification of those at high risk for age-related diseases.

## Supplementary Information

Additional file 1.

## Data Availability

All data used for this analysis is publicly available from the NHANES website (https://www.cdc.gov/nchs/nhanes/index.htm).

## Appendix

See Table 1
