# Supplementary material for: Timing of menarche and menopause and epigenetic aging among U.S. adults: results from the National Health and Nutrition Examination Survey 1999–2002
Source: Clin Epigenetics. 2025 Feb 21;17:31. doi: 10.1186/s13148-025-01827-x (PMC11844159; doi:10.1186/s13148-025-01827-x)
Supplement: Supplementary file 1 — Additional file 1. [file 13148_2025_1827_MOESM1_ESM.pdf]

Supplemental Methods

Age at Menarche and Menopause

Age at menarche was obtained by asking participants, "How old were you when you had your first menstrual period?" (Reproductive Health Questionnaire, RHQ010).(1,2)

Age at menopause was assessed through a series of questions in the RHQ.(1,2) Participants were first asked "Have you had regular periods in the last 12 months?" (RHQ030). Those answering "No" due to medical conditions/treatment or usual irregularity (N=109), "Yes" (N=17), or unknown period regularity (N=1) were excluded (RHQ040). Participants who had their last menstrual period <12 months since the time of screening (N=43) (RHQ050) and those who did not know the time elapsed since their last menstrual period (N=1, RHQ050) or age at last period (N=75, RHQ060) were further excluded. Finally, those reporting menopause before age 40 (N=174) or after age 62 years (N=4) were removed since pathologic conditions may have influenced the occurrence of menopause at such ages.(3) Age at menopause was then calculated as 12 months after age at last menstrual period for those remaining in the sample.

DNA Methylation and Epigenetic Age

Epigenetic age measures and DNA methylation (DNAm)-based leukocyte proportion estimates were downloaded from the NHANES website.(4) DNA was extracted from whole blood of a subset of NHANES participants aged ≥50 years old from the 1999-2000 and 2000-2001 survey cycles. Genome-wide DNAm was measured using the Illumina EPIC BeadChip array. Quality control steps and DNAm data processing were completed, as described elsewhere.(4) The Horvath,(5) Hannum,(6) SkinBlood,(7) PhenoAge,(8) GrimAge,(9) GrimAge2,(10) DNAmTL,(11) and DunedinPoAm(12) epigenetic age biomarkers were included in analyses. Pearson correlation coefficients and median absolute error (MAE) were used to assess the fit for each epigenetic age biomarker and chronological age.

Statistical Analysis

Generalized linear regression models were run using the *svyglm* function from the R 'Survey' package(13) to account for survey weights, correcting for selection, non-response, and coverage biases, following NHANES analytic guidelines for the epigenetic clock sample. Potential confounders/model covariates were identified *a priori* and included chronological age in years (continuous), chronological age in years squared (continuous), and self-identified race/ethnicity (Non-Hispanic White, Mexican American, Other Hispanic, Non-Hispanic Black, Other Race - Including Multi-Racial).

Sensitivity analyses

We further adjusted models for the original covariate set plus (i) body mass index (BMI) (continuous), smoking status (ever, never), and alcohol intake (abstainer, moderate, heavy drinker) at the time of the NHANES screening and (ii) estimated cell-type proportions (CD8 cells, CD4 cells, NK cells, B cells, monocytes, and neutrophils).

Given the wide age range of participants at blood draw (50-84 years), we considered chronological age as an effect modifier in the relationship between age at menarche and age at menopause with epigenetic aging. We conducted analyses stratified by chronological age (<65 years vs. 65-84 years) and fit statistical interaction terms between chronological age and each exposure.

Finally, given the main epigenetic age results, we evaluated associations of age at menopause with the DNA methylation components of GrimAge and GrimAge2 — smoking pack-years, adrenomedullin (ADM), beta-2 microglobulin (B2M), cystatin C, growth differentiation factor 15 (GDF15), leptin, plasminogen activation inhibitor 1 (PAI1), tissue inhibitor metalloproteinase 1 (TIMP1), C-reactive protein (CRP), and hemoglobin A1C.(9,10) We report adjusted regression coefficients (B) and 95% Confidence Intervals (CIs) to evaluate statistical significance and precision. All statistical analyses were performed using R version 4.3.1.(14)

References

1. RHQ [Internet]. [cited 2024 Dec 11]. Available from: https://wwwn.cdc.gov/Nchs/Data/Nhanes/Public/1999/DataFiles/RHQ.htm

2. RHQ_B [Internet]. [cited 2024 Dec 11]. Available from: https://wwwn.cdc.gov/Nchs/Data/Nhanes/Public/2001/DataFiles/RHQ_B.htm

3. Costanian C, McCague H, Tamim H. Age at natural menopause and its associated factors in Canada: cross-sectional analyses from the Canadian Longitudinal Study on Aging. Menopause. 2018 Mar;25(3):265.

4. NHANES 1999-2002 DNA Methylation Array and Epigenetic Biomarkers [Internet]. [cited 2024 Nov 28]. Available from: https://wwwn.cdc.gov/nchs/nhanes/dnam/

5. Horvath S. DNA methylation age of human tissues and cell types. Genome Biology. 2013 Dec 10;14(10):3156.

6. Hannum G, Guinney J, Zhao L, Zhang L, Hughes G, Sadda S, et al. Genome-wide Methylation Profiles Reveal Quantitative Views of Human Aging Rates. Molecular Cell. 2013 Jan 24;49(2):359–67.

7. Horvath S, Oshima J, Martin GM, Lu AT, Quach A, Cohen H, et al. Epigenetic clock for skin and blood cells applied to Hutchinson Gilford Progeria Syndrome and ex vivo studies. Aging (Albany NY). 2018 Jul 26;10(7):1758–75.

8. Levine ME, Lu AT, Quach A, Chen BH, Assimes TL, Bandinelli S, et al. An epigenetic biomarker of aging for lifespan and healthspan. Aging (Albany NY). 2018 Apr 17;10(4):573–91.

9. Lu AT, Quach A, Wilson JG, Reiner AP, Aviv A, Raj K, et al. DNA methylation GrimAge strongly predicts lifespan and healthspan. Aging (Albany NY). 2019 Jan 21;11(2):303–27.

10. Lu AT, Binder AM, Zhang J, Yan Q, Reiner AP, Cox SR, et al. DNA methylation GrimAge version 2. Aging (Albany NY). 2022 Dec 14;14(23):9484–549.

11. Lu AT, Seeboth A, Tsai PC, Sun D, Quach A, Reiner AP, et al. DNA methylation-based estimator of telomere length. Aging (Albany NY). 2019 Aug 18;11(16):5895–923.

12. Belsky DW, Caspi A, Arseneault L, Baccarelli A, Corcoran DL, Gao X, et al. Quantification of the pace of biological aging in humans through a blood test, the DunedinPoAm DNA methylation algorithm. Hagg S, Tyler JK, Hagg S, Justice J, Suderman M, editors. eLife. 2020 May 5;9:e54870.

13. R: Survey-weighted generalised linear models. [Internet]. [cited 2024 Dec 11]. Available from: https://r-survey.r-forge.r-project.org/survey/html/svyglm.html

14. R: The R Project for Statistical Computing [Internet]. [cited 2024 Dec 11]. Available from: https://www.r-project.org/

Supplemental Figure 1: Flowchart of menarche analysis participants (N=1,033).

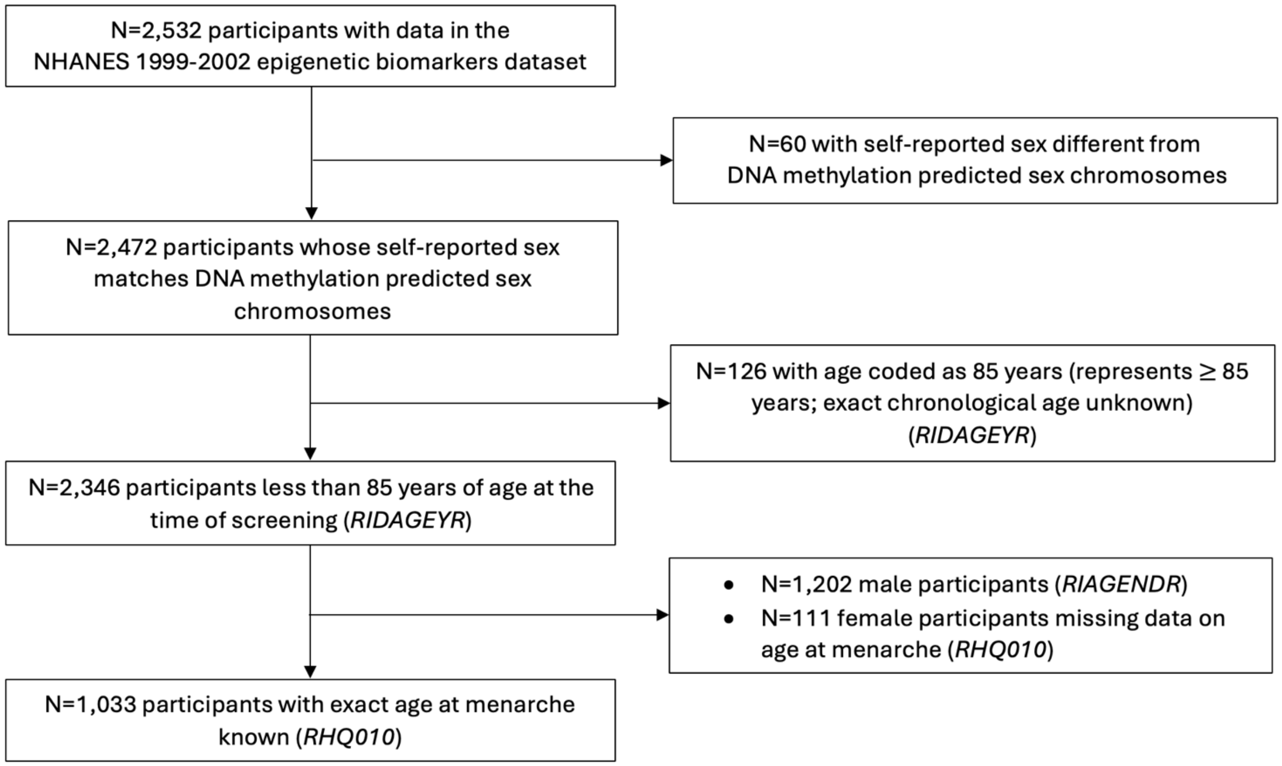

<u>Supplemental Figure 2</u>: Flowchart of menopause analysis participants (N=658).

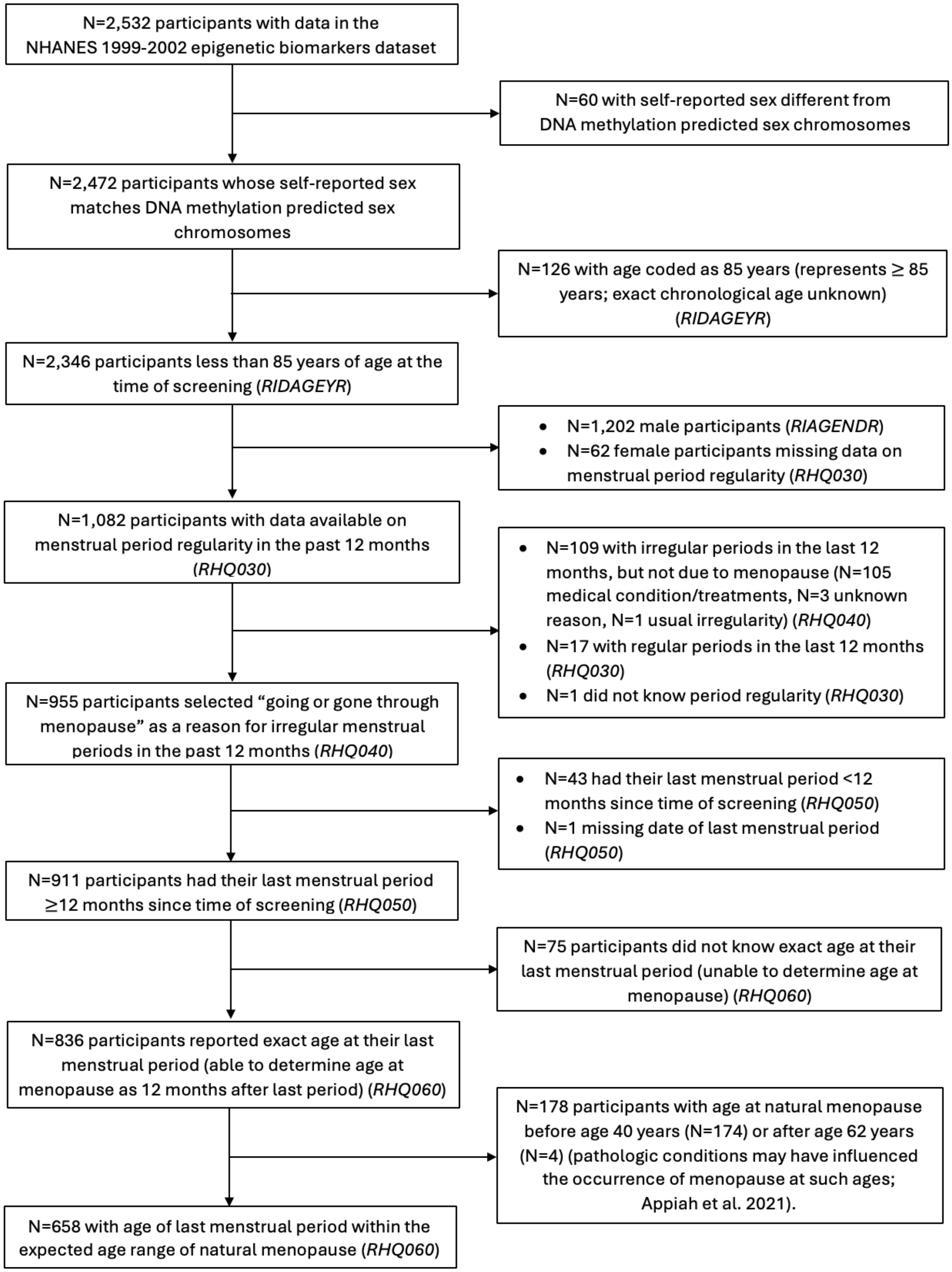

<u>Supplemental Figure 3</u>: Epigenetic clock performance in total sample (both menarche and menopause analyses) (N=1,045).

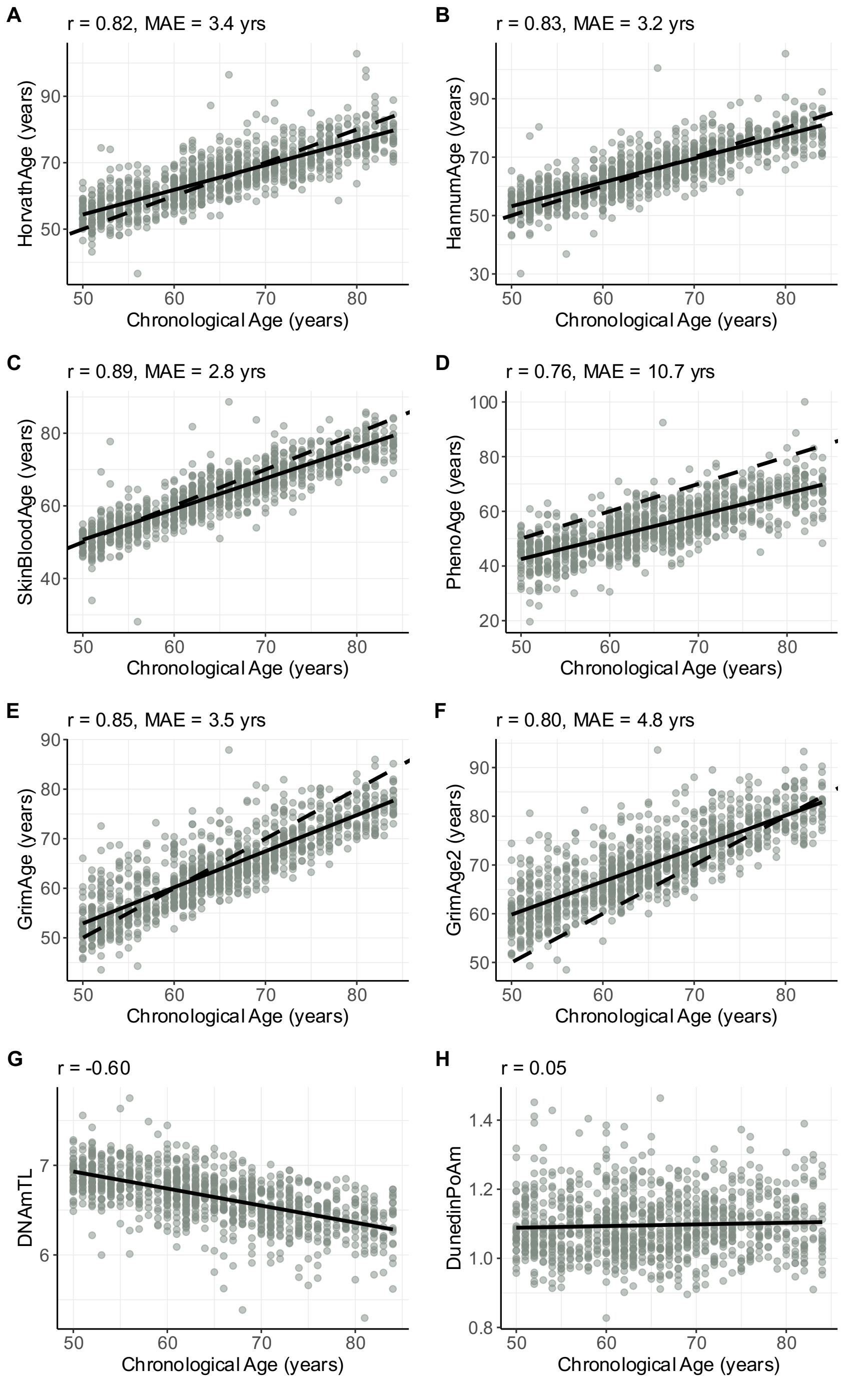

Pearson correlation coefficient *r* and median absolute error (MAE) between chronological age based on birth date and epigenetic age estimated by each epigenetic clock.

The linear trendline and 95% CI are plotted as a solid line with shaded area and the identity line (y=x) is plotted as a dashed line.

<u>Supplemental Figure 4</u>: Associations of DNAmTL and DunedinPoAm with (a) age at menarche and (b) age at menopause.

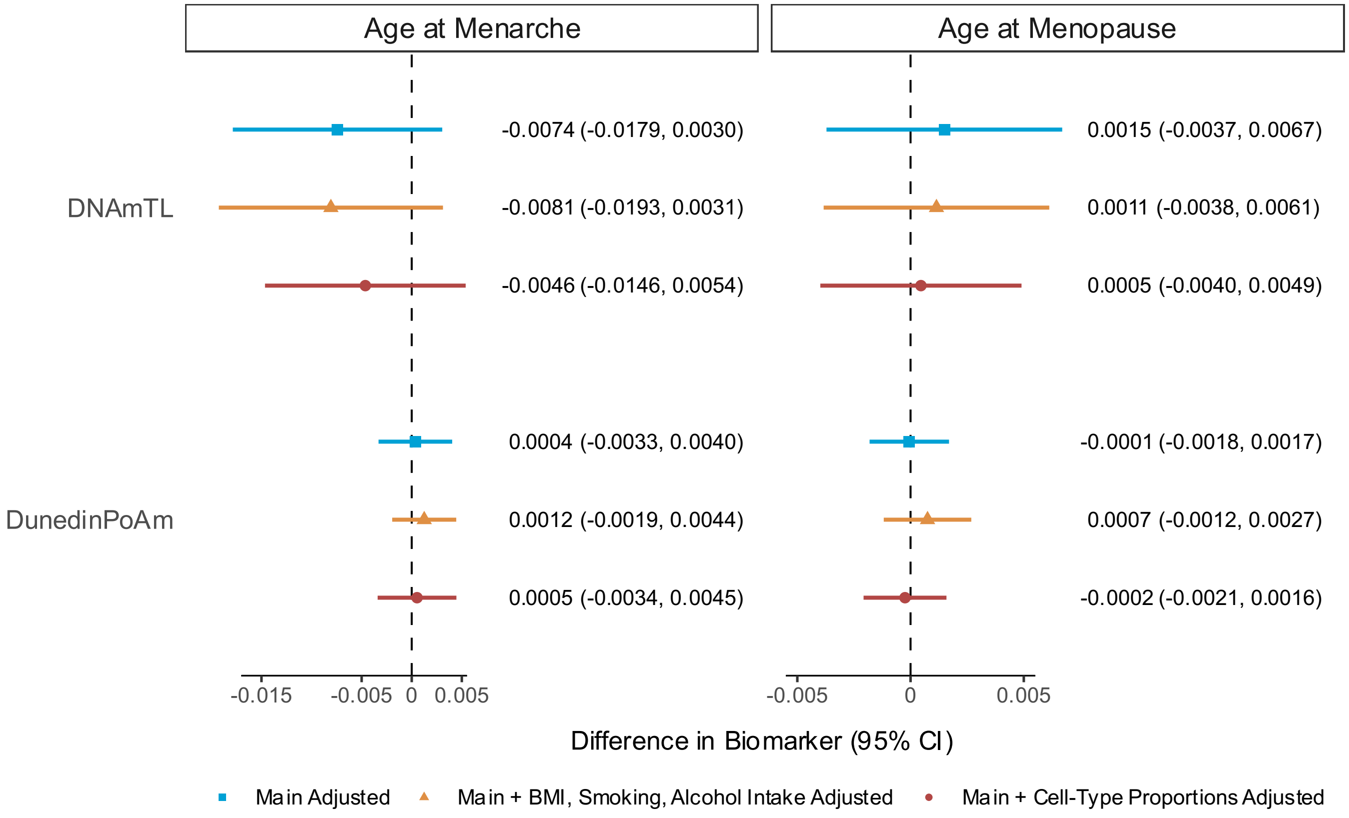

Associations were assessed using survey-weighted generalized linear regression models. Main models (blue) were adjusted for chronological age in years (continuous), chronological age squared, and self-identified race/ethnicity. In sensitivity analyses, models were adjusted for covariates in main models in addition to i) BMI, smoking status, and alcohol intake at NHANES screening (yellow) and ii) cell-type proportions (red).

Estimates are regression coefficients (B) that represent the estimated change in each biomarker per one-year increase in age at menarche or age at menopause.

Supplemental Figure 5: Associations of epigenetic age deviation with (a) age at menarche and (b) age at menopause (additionally adjusted for BMI, smoking, and alcohol intake).

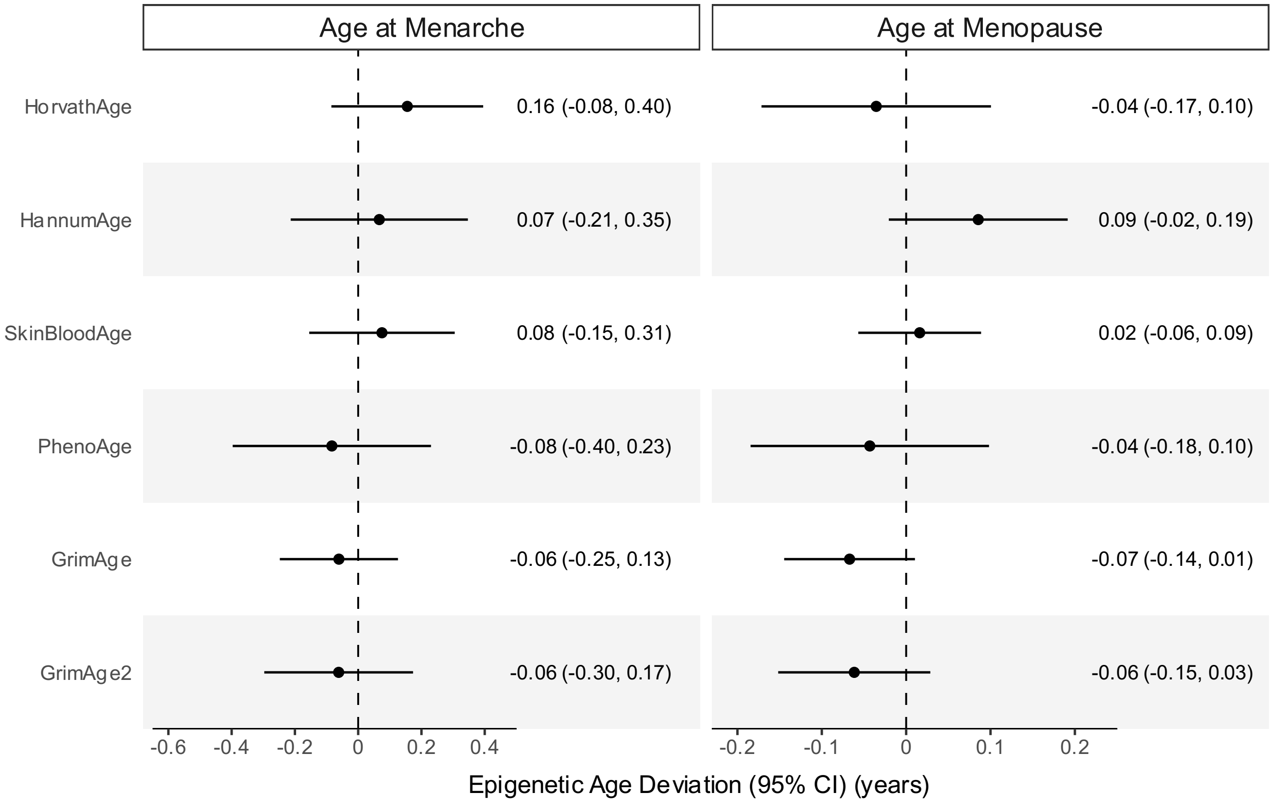

Associations were assessed using survey-weighted generalized linear regression models. All models were adjusted for chronological age in years (continuous), chronological age squared, self-identified race/ethnicity, body mass index at time of screening, smoking status at time of screening, and alcohol intake at time of screening.

Estimates are regression coefficients (B) that represent the estimated change in each epigenetic age biomarker per one-year increase in age at menarche or age at menopause.

<u>Supplemental Figure 6</u>: Associations of epigenetic age deviation with (a) age at menarche and (b) age at menopause (additionally adjusted for cell-type proportions).

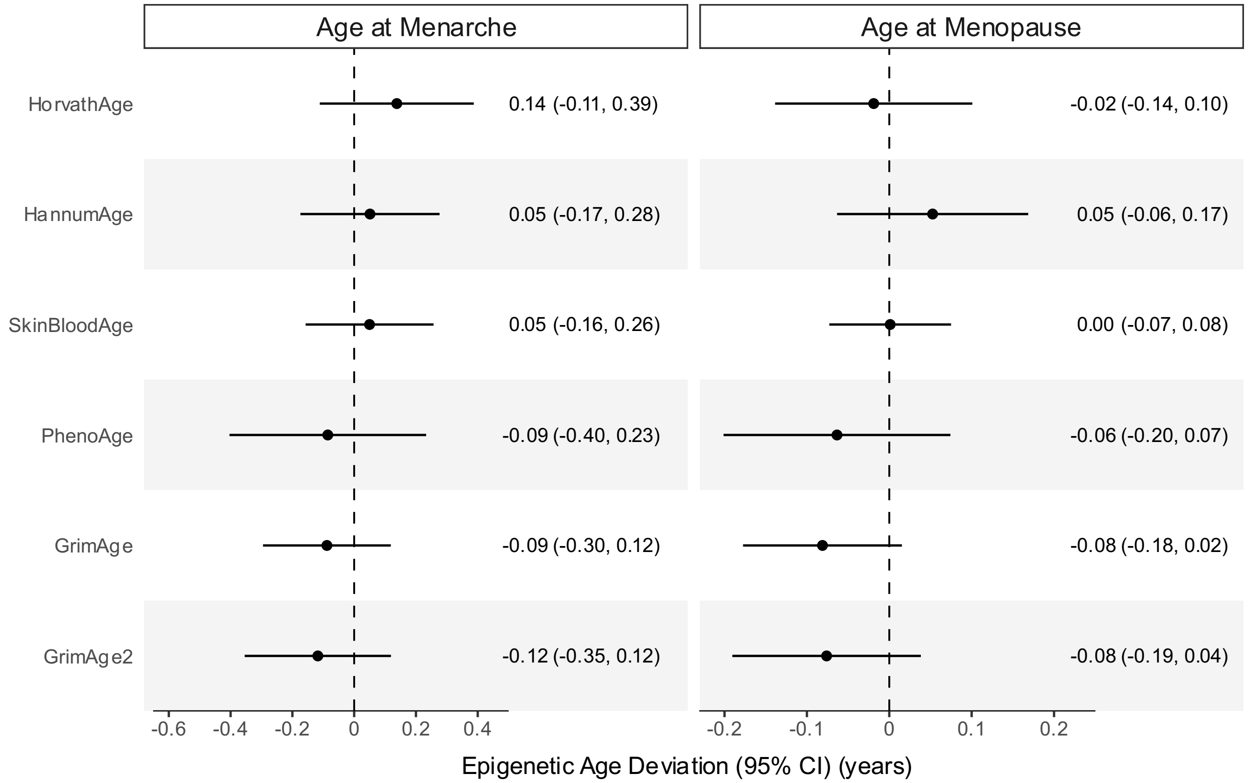

Associations were assessed using survey-weighted generalized linear regression models. All models were adjusted for chronological age in years (continuous), chronological age squared, self-identified race/ethnicity, and estimated cell-type proportions (CD8 cells, CD4 cells, NK cells, B cells, monocytes, and neutrophils).

Estimates are regression coefficients (B) that represent the estimated change in each epigenetic age biomarker per one-year increase in age at menarche or age at menopause.
